# Efficacy and Safety of Ritlecitinib in the Asian Subpopulation of the ALLEGRO‐2b/3 and ALLEGRO‐LT Clinical Studies for Alopecia Areata

**DOI:** 10.1111/1346-8138.70154

**Published:** 2026-02-06

**Authors:** Rie Ueki, Masato Mizuashi, Kazutoshi Harada, Xingqi Zhang, Wenyu Wu, Wen‐Hung Chung, Ohsang Kwon, Xin Luo, Victoria Basey, Robert Wolk, Nanzhi Shi, Kayo Fujita, Yimeng Shen, Tomohiro Hirose

**Affiliations:** ^1^ Juntendo University Tokyo Japan; ^2^ Tohoku University Graduate School of Medicine Sendai Japan; ^3^ Department of Dermatology Tokyo Medical University Tokyo Japan; ^4^ The First Affiliated Hospital, Sun Yat‐Sen University Guangzhou China; ^5^ Huashan Hospital Affiliated to Fudan University Shanghai China; ^6^ Department of Dermatology Chang Gung Memorial Hospital Taipei Taiwan; ^7^ Department of Dermatology Seoul National University College of Medicine Seoul South Korea; ^8^ Pfizer R&D China Shanghai China; ^9^ Pfizer Inc Tadworth UK; ^10^ Pfizer Inc Groton Connecticut USA; ^11^ Pfizer R&D Japan G.K. Tokyo Japan; ^12^ Pfizer China PBG Beijing China; ^13^ Pfizer Japan Inc Tokyo Japan

**Keywords:** alopecia areata, Asian people, east Asian people, hair disease, Janus kinase inhibitors

## Abstract

Ritlecitinib is an oral JAK3/TEC family kinase inhibitor approved for individuals aged ≥ 12 years with severe alopecia areata. Here we report interim efficacy and safety results with ritlecitinib up to Month 24 in the Asian subpopulation from the ALLEGRO phase 2b/3 and ALLEGRO‐LT studies. Data are reported for participants with Severity of Alopecia Tool score ≥ 50 who received ritlecitinib 50 mg with (200/50‐mg group) or without (50‐mg group) an initial 4‐week 200‐mg daily loading dose in ALLEGRO‐2b/3, and continued ritlecitinib 50 mg in ALLEGRO‐LT. In long‐term studies, it is common for summaries at later time points to reflect participants who remained in the study, tolerated the drug, and had a positive treatment response. To account for this, efficacy summaries were presented both as observed and by using the last observation carried forward method. At Month 12, 51.7% and 44.7% (last observation carried forward) of Asian participants in the 50‐mg (*n* = 58) and 200/50‐mg groups (*n* = 47), respectively, achieved Severity of Alopecia Tool score ≤ 20 (≤ 20% scalp hair loss); the respective proportions were 50.0% and 47.8% at Month 24. Of participants who achieved Severity of Alopecia Tool score ≤ 20 at Month 12 in the 50‐mg and 200/50‐mg groups, 68.2% and 86.7% sustained this response through Month 24, respectively. At Month 12, 74.0% and 57.9% of participants in the 50‐mg and 200/50‐mg groups, respectively, had an eyebrow hair regrowth response; at Month 24, the proportions were 72.0% and 48.7%. At Month 12, 65.1% and 54.1% of participants in the 50‐mg and 200/50‐mg groups, respectively, had an eyelash hair regrowth response; the proportions were 65.1% and 52.8% at Month 24. The proportion of participants with a Patients' Global Impression of Change response of “moderately” or “greatly” improved since baseline increased through Month 24 in both groups. Ritlecitinib had an acceptable safety profile, consistent with that in the overall population. Overall, ritlecitinib 50 mg (±200‐mg loading dose) demonstrated clinically meaningful and sustained efficacy through Month 24 in the Asian subpopulation, consistent with results in the overall population.

**Trial Registration:** ClinicalTrials.gov: NCT04006457.

## 1 Introduction

Alopecia areata (AA) is an autoimmune disease that has an underlying immunoinflammatory pathogenesis and is characterized by nonscarring hair loss of the scalp, face, and/or body [1]. AA can present as patchy hair loss, complete scalp hair loss (alopecia totalis), or total loss of hair on the scalp and body (alopecia universalis). AA has an estimated prevalence of 0.58%–2% globally [2, 3, 4, 5]; however, data are limited on the prevalence of AA in Asian countries. Prior studies have estimated the prevalence to be 0.27%–2.5% in Japan [6, 7] and 0.24% across six provinces in China [8]. Most studies have suggested no significant racial predisposition to AA [9, 10, 11, 12]; however, one study found a three‐fold higher incidence of AA in people of Asian origin [13].

Baricitinib, an oral Janus kinase (JAK) 1/2 inhibitor, is approved for the treatment of adults with severe AA in the US, Japan, EU, China, and several other countries [14]. Deuruxolitinib, another oral JAK1/2 inhibitor, is approved to treat severe AA in adults in the US [15]. Ritlecitinib 50 mg once daily (QD), an oral JAK3/TEC family kinase inhibitor, is approved to treat adults and adolescents aged ≥ 12 years with severe AA in the US, Japan, EU, China, and several other countries [16].

In the ALLEGRO phase 2b/3 study (ALLEGRO‐2b/3; NCT03732807) ritlecitinib demonstrated efficacy and acceptable safety in participants aged ≥ 12 years with AA over 48 weeks [17]. Results in the Asian subpopulation treated with ritlecitinib in the ALLEGRO‐2b/3 study were consistent with those in the overall population [18]. ALLEGRO‐LT (NCT04006457) is an ongoing, phase 3, open‐label study investigating the long‐term safety and efficacy of ritlecitinib in AA [19, 20]. This subgroup analysis aimed to report updated interim efficacy and safety results with ritlecitinib in the Asian subpopulation up to Month 24 in the ALLEGRO‐2b/3 and ALLEGRO‐LT studies.

## 2 Methods

### 2.1 Study Design and Participants

ALLEGRO‐2b/3 was a randomized, double‐blind, multicenter clinical trial in which participants were randomized to receive ritlecitinib 30 or 50 mg QD with or without an initial 4‐week 200‐mg QD loading dose, ritlecitinib 10 mg (included only for dose–response evaluation), or placebo for 24 weeks [17]. During a subsequent 24‐week extension period, ritlecitinib groups continued on their maintenance dose (50, 30, or 10 mg), and those initially randomized to placebo switched to ritlecitinib 50 mg QD with or without a 4‐week 200‐mg QD loading dose.

ALLEGRO‐LT is an ongoing study that includes rollover participants who received ritlecitinib in the ALLEGRO‐2a (NCT02974868) and ALLEGRO‐2b/3 clinical trials, as well as de novo participants with ≥ 25% scalp hair loss who had not received ritlecitinib treatment in either study. Rollover participants received ritlecitinib 50 mg QD, and de novo participants received ritlecitinib 200 mg QD for 4 weeks followed by 50 mg QD for up to 60 additional months [20, 21].

**FIGURE 1:**
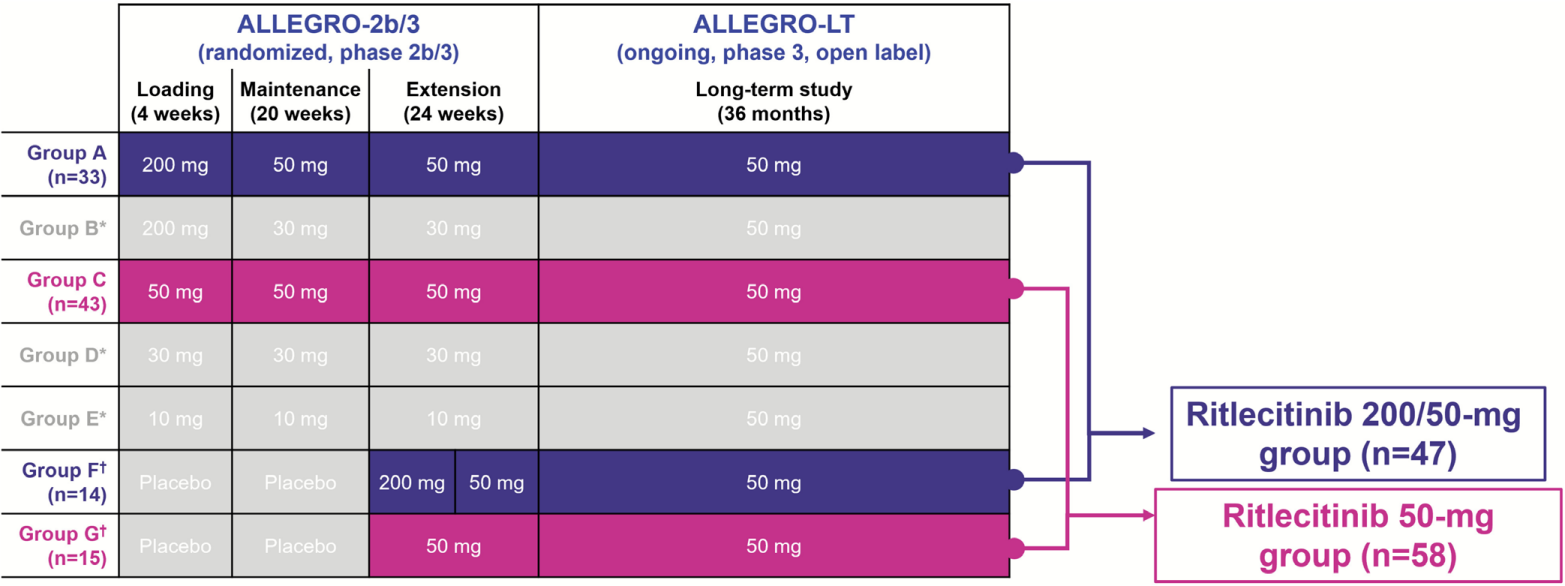
Participants from ALLEGRO‐2b/3 and ALLEGRO‐LT included in the Asian subpopulation analysis. *Data from participants in groups B, D, and E were not included in this analysis. **Data from participants in groups F and G while on placebo were not included in this analysis; data from participants in groups F and G were re‐baselined from the start of treatment with ritlecitinib.

This subgroup analysis included Asian participants (defined as those of Asian descent) from ALLEGRO‐2b/3. After completing ALLEGRO‐2b/3, patients could roll over into ALLEGRO‐LT (Figure 1). In this analysis, patients in the 200/50‐mg group received either an initial 4‐week loading dose of ritlecitinib 200 mg QD followed by ritlecitinib 50 mg for 44 weeks, or placebo for 24 weeks followed by a 4‐week ritlecitinib 200‐mg QD loading dose then ritlecitinib 50 mg QD for 20 weeks in ALLEGRO‐2b/3. Patients who initially received placebo were re‐baselined, as discussed below. Patients in the 50‐mg group in this analysis received either ritlecitinib 50‐mg QD dose for 48 weeks, or placebo for 24 weeks followed by ritlecitinib 50 mg QD for 24 weeks in ALLEGRO‐2b/3. Patients who initially received placebo were re‐baselined, as discussed below. Both groups continued to receive ritlecitinib 50 mg QD in ALLEGRO‐LT. The 30‐mg and 10‐mg treatment groups in the ALLEGRO‐2b/3 study were not included in this analysis.

Here we present results reported up to the cutoff date of December 9, 2022 (database not locked; data may change).

Written informed consent was obtained from each participant or participant's parent or legal representative.

### 2.2 Analysis Populations

The key inclusion criteria in ALLEGRO‐2b/3 were age of ≥ 12 years, a diagnosis of AA with ≥ 50% scalp hair loss due to AA (including alopecia totalis and alopecia universalis), no evidence of terminal hair regrowth within 6 months at both screening and baseline visits, and a maximum duration of current hair loss episode of ≤ 10 years [17].

Participants who rolled over from ALLEGRO‐2b/3 into ALLEGRO‐LT had to have completed ≥ 34 weeks of study intervention in ALLEGRO‐2b/3. Adolescents in ALLEGRO‐LT were required to have an improvement in Severity of Alopecia Tool (SALT) score of ≥ 50% from baseline (in ALLEGRO‐2b/3) at Month 3 and a SALT score of ≤ 20 by Month 6 to continue in ALLEGRO‐LT.

This subgroup analysis included patients who self‐identified as Asian (defined as patients of Asian descent).

All analyses were descriptive and exploratory and conducted without formal hypothesis testing.

### 2.3 Re‐Baselining

Among the patients who rolled over from ALLEGRO‐2b/3 into ALLEGRO‐LT, a portion received placebo during the initial 24 weeks of the study. In order to allow for analysis of all patients within the ALLEGRO‐LT study concurrently, patients who initially received placebo were re‐baselined to align the start of ritlecitinib treatment and time points within and across groups (Figure 1). All data are reported in months; 4 weeks in ALLEGRO‐2b/3 was considered equivalent to 1 month in ALLEGRO‐LT.

### 2.4 Efficacy Measures

For this subgroup analysis, efficacy data are presented up to Month 24 for the two groups (i.e., the 200/50‐mg and 50‐mg groups). The clinician‐reported outcomes included the proportion of participants with response through Month 24 based on a SALT score of ≤ 20 and ≤ 10. SALT assesses the amount of scalp hair loss and has scores ranging from 0 (no scalp hair loss) to 100 (complete scalp hair loss).

In participants with abnormal eyebrow assessment (EBA) and eyelash assessment (ELA) scores at baseline, the proportion of participants achieving EBA and ELA responses were analyzed based on a ≥ 2‐grade improvement from baseline or achieving a normal score (3). EBA and ELA are four‐point scales ranging from 0 (none, or no eyebrows/eyelashes) to 3 (normal eyebrows/eyelashes).

The participant‐reported outcome endpoints included the proportion of participants achieving a Patients' Global Impression of Change (PGI‐C) score of “moderately improved” or “greatly improved” from baseline. PGI‐C is a self‐reported single‐item scale on which participants rate the improvement or worsening of AA symptoms compared with status at the start of the study; the scale has seven responses ranging from “greatly improved” to “greatly worsened.”

In addition, the proportion of participants with a sustained SALT ≤ 20 response (participants with a SALT ≤ 20 response at Months 12 and 24 who did not have a SALT score of > 20 at any in‐between time point) and the proportion with a maintained SALT ≤ 20 response (participants with a SALT ≤ 20 response at Months 12 and 24, regardless of SALT scores at in‐between time points) were assessed.

### 2.5 Safety Assessments

Adverse events (AEs), serious AEs (SAEs), and events of interest (i.e., opportunistic infections, cardiovascular and malignancy events) were monitored throughout the study. The events of interest were reviewed by adjudication committees using predefined criteria (MedDRA v27.0).

### 2.6 Statistical Analysis

Efficacy data are presented through Month 24 as observed data and as imputed data (last observation carried forward [LOCF]) to account for missing data values. LOCF was applied to each visit for all participants with missing data, except for those who had not yet reached that analysis visit.

These interim, pooled analyses are descriptive in nature, and formal hypothesis testing was not conducted. The 95% confidence intervals were calculated based on normal approximation. Safety data are reported descriptively.

## 3 Results

### 3.1 Participant Disposition

The Asian subpopulation included 105 participants, with 58 in the 50‐mg group and 47 in the 200/50‐mg group. At the data cutoff date, 37 (63.8%) and 34 (72.3%) participants, respectively, continued to receive ritlecitinib and were undergoing assessments (Table S1). Baseline characteristics were generally similar between the two treatment groups (Table 1). The mean (SD) age was 31.1 (10.9) years and 30.4 (11.8) years in the 50‐mg and 200/50‐mg groups, respectively. The mean (SD) duration of AA since diagnosis was 7.2 (7.0) years and 7.5 (5.6) years in the 50‐mg and 200/50‐mg groups, respectively. Mean (SD) duration of current AA episode was 3.5 (2.8) years and 3.5 (2.9) years in the 50‐mg and 200/50‐mg groups, respectively.

**TABLE 1:** Baseline characteristics of the Asian subpopulation.

	Ritlecitinib, 50 mg (*n* = 58)	Ritlecitinib, 200/50 mg (*n* = 47)
Age		
Mean (SD), years	31.1 (10.9)	30.4 (11.8)
12–17 years, *n* (%)	3 (5.2)	5 (10.6)
≥ 18 years, *n* (%)	55 (94.8)	42 (89.4)
Female, *n* (%)	30 (51.7)	31 (66.0)
AT/AU, *n* (%)*	25 (43.1)	23 (48.9)
Baseline SALT score, mean (SD)	89.4 (15.2)	92.7 (12.9)
Duration of AA since diagnosis, mean (SD), years	7.2 (7.0)	7.5 (5.6)
Duration of current AA episode, mean (SD), years	3.5 (2.8)	3.5 (2.9)

Abbreviations: AA, alopecia areata; AT, alopecia totalis; AU, alopecia universalis; SALT, Severity of Alopecia Tool; SD, standard deviation.

* Participants in the AT/AU category had a SALT score of 100 at baseline (regardless of the category in the AA history case report form).

SALT scores at baseline and at the last visit of the ALLEGRO‐2b/3 study for participants who completed ALLEGRO‐2b/3 but did not roll over into ALLEGRO‐LT are summarized in Table S2.

### 3.2 Efficacy

**FIGURE 2:**
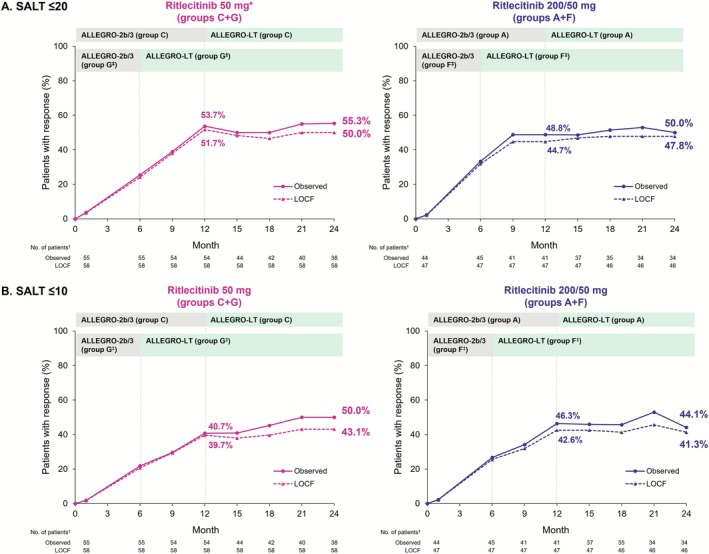
Proportion of participants with (A) a SALT score of ≤ 20 and (B) a SALT score of ≤ 10 through Month 24 in the Asian subpopulation. AA, alopecia areata; LOCF, last observation carried forward; SALT, Severity of Alopecia Tool. *Ritlecitinib 50 mg daily is the approved dose in Japan. ^†^Number of participants with valid data at that analysis visit. ^‡^Data while participants were on placebo were not included in this analysis; data from participants in groups F and G were re‐baselined from the start of treatment with ritlecitinib.

**FIGURE 3:**
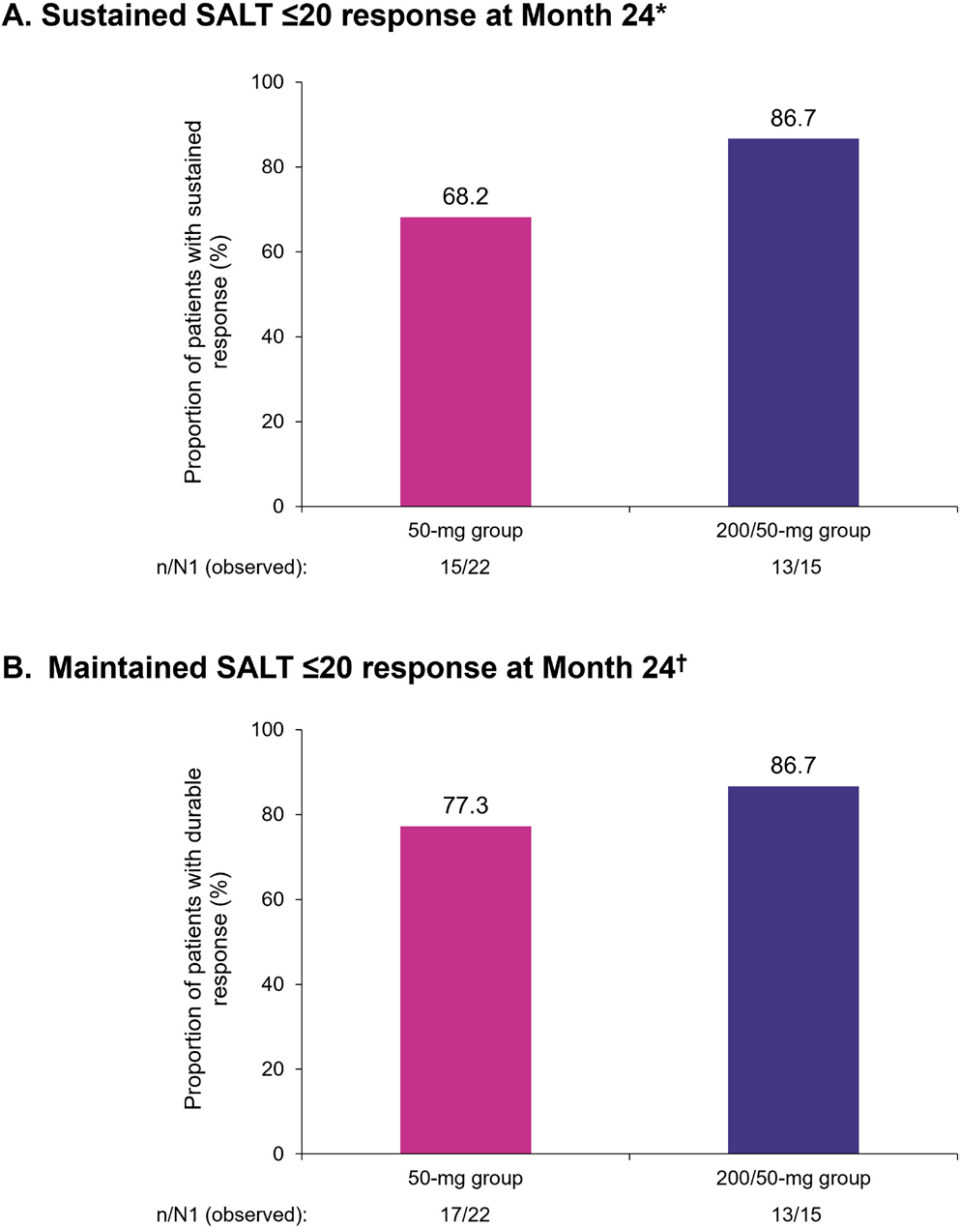
Proportion of participants with (A) sustained SALT ≤ 20 response at Month 24 among those with SALT ≤ 20 response at Month 12* in the Asian subpopulation and (B) maintained SALT ≤ 20 response at Month 24 among those with SALT < 20 response at Month 12 in the Asian subpopulation^†^. SALT, Severity of Alopecia Tool. Percentages are based on n/N1 where *n* = the number of participants with sustained response and N1 = total number of participants with the observed value at Month 24. Observed data are presented. *Sustained SALT ≤ 20 response was defined as having a SALT score of ≤ 20 at Months 12 and 24 and not having a SALT score of > 20 at any time point between Months 12 and 24. ^**†**^Maintained SALT ≤ 20 response was defined as having a SALT score of ≤ 20 at Months 12 and 24, regardless of whether the patient had a SALT score of > 20 at any time point between them.

The proportion of participants with a SALT ≤ 20 response remained consistent through Month 24 in both groups. At Month 12, 53.7% and 48.8% (observed) and 51.7% and 44.7% (LOCF) of participants in the 50‐mg and 200/50‐mg groups, respectively, had SALT scores of ≤ 20; the proportions were 55.3% and 50.0% (observed) and 50.0% and 47.8% (LOCF), respectively, at Month 24 (Figure 2A). The proportion of participants with a SALT ≤ 10 response also remained consistent through Month 24 in both groups. At Month 12, 40.7% and 46.3% (observed) and 39.7% and 42.6% (LOCF) of participants in the 50‐mg and 200/50‐ mg groups, respectively, had a SALT score of ≤ 10; the proportions were 50.0% and 44.1% (observed) and 43.1% and 41.3% (LOCF), respectively, at Month 24 (Figure 2B). Of SALT ≤ 20 responders at Month 12, 68.2% (50 mg) and 86.7% (200/50 mg) sustained this response through Month 24 (observed) (Figure 3A). Maintenance of SALT ≤ 20 response at Month 24 among those with a SALT ≤ 20 response at Month 12 was 77.3% (50 mg) and 86.7% (200/50 mg), respectively (observed) (Figure 3B).

**FIGURE 4:**
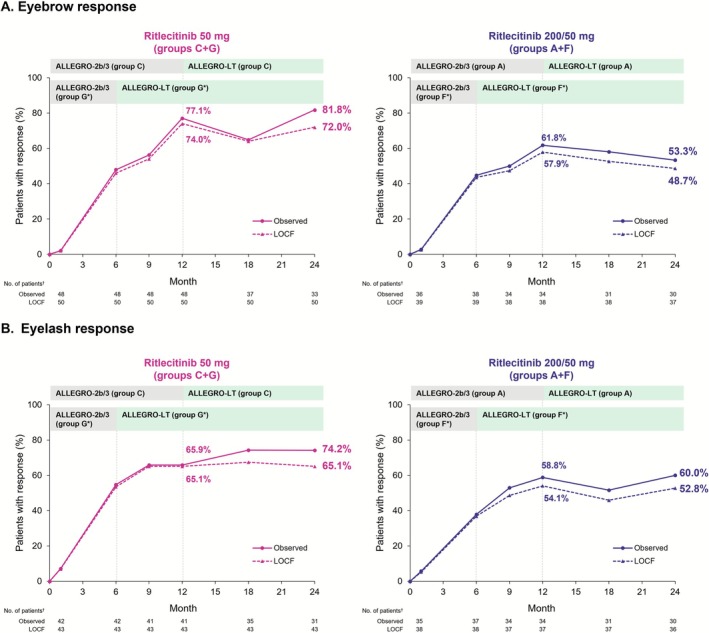
Proportions of participants with (A) eyebrow response (≥ 2‐grade improvement or normal EBA score) and (B) eyelash response (≥ 2‐grade improvement or normal ELA score) from baseline to Month 24 in the Asian subpopulation. (A) Eyebrow response. (B) Eyelash response. EBA, eyebrow assessment; ELA, eyelash assessment; LOCF, last observation carried forward. *Data while the participants were on placebo were not included in this analysis; data from participants in groups F and G were re‐baselined from the start of treatment with ritlecitinib. ^†^Number of participants with valid data at that analysis visit.

The proportion of participants with EBA response among those with an abnormal EBA score at baseline remained stable through Month 24 in both treatment groups. At Month 12, 77.1% and 61.8% (observed) and 74.0% and 57.9% (LOCF) of participants in the 50‐mg and 200/50‐mg groups, respectively, had an EBA response; the proportions were 81.8% and 53.3% (observed) and 72.0% and 48.7% (LOCF), respectively, at Month 24 (Figure 4A). The proportion of participants with ELA response among those with an abnormal ELA score at baseline also remained stable through Month 24 in both treatment groups. At Month 12, 65.9% and 58.8% (observed) and 65.1% and 54.1% (LOCF) of participants in the 50‐mg and 200/50‐mg groups, respectively, had an ELA response; the proportions were 74.2% and 60.0% (observed) and 65.1% and 52.8% (LOCF), respectively, at Month 24 (Figure 4B).

**FIGURE 5:**
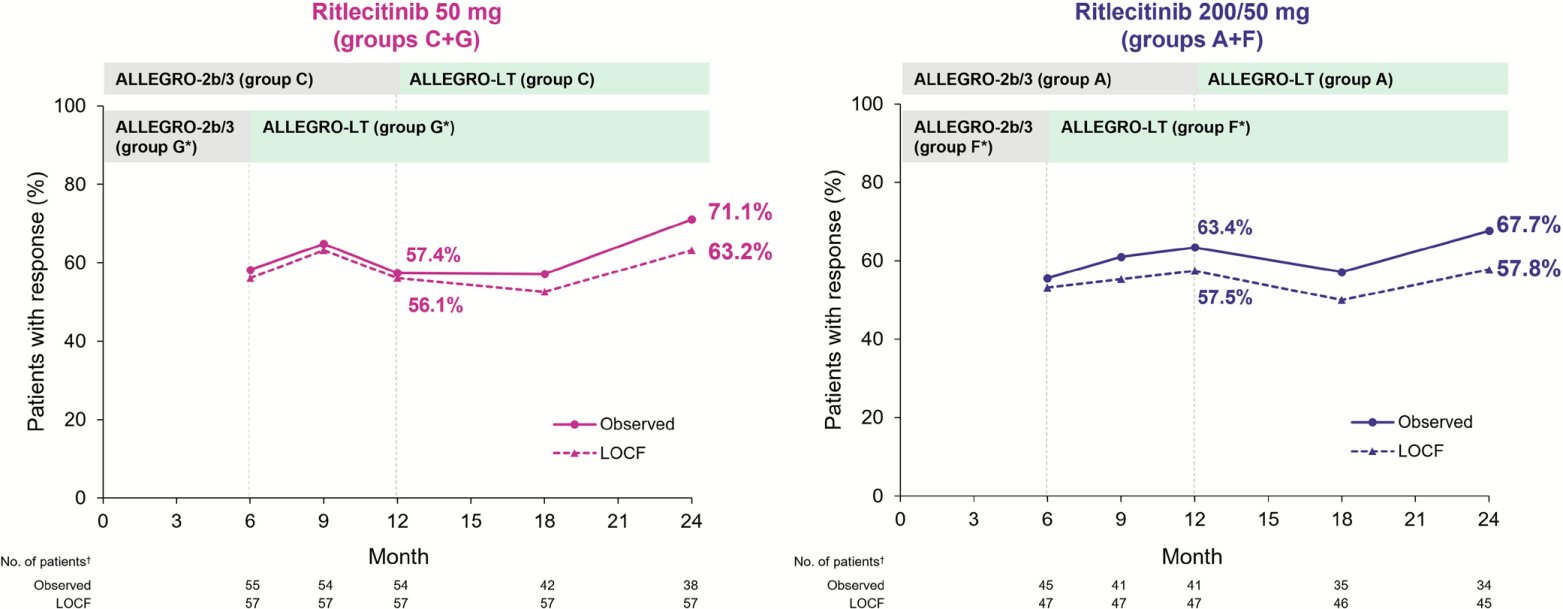
Proportions of participants with PGI‐C response* from baseline to Month 24 in the Asian subpopulation in ALLEGRO‐2b/3 and ALLEGRO‐LT clinical studies for AA. AA, alopecia areata; LOCF, last observation carried forward; PGI‐C, Patients' Global Impression of Change. PGI‐C response = score of “moderately” or “greatly” improved from baseline. The PGI‐C asks the participant to evaluate the improvement or worsening of AA symptoms compared with status at the start of the study. *Data while the participants were on placebo were not included in this analysis; data from participants in groups F and G were re‐baselined from the start of treatment with ritlecitinib. ^†^Number of participants with valid data at that analysis visit.

At Month 12, PGI‐C response rates were 57.4% (observed) and 56.1% (LOCF) in the 50‐mg group and 63.4% (observed) and 57.5% (LOCF) in the 200/50‐mg group. At Month 24, the response rates were 71.1% (observed) and 63.2% (LOCF), respectively, in the 50‐mg group and 67.6% (observed) and 57.8% (LOCF) in the 200/50‐mg group (Figure 5).

### 3.3 Safety

In the subgroup of Asian participants, 57 participants (98.3%) in the 50‐mg group and 44 (93.6%) in the 200/50‐mg group experienced at least one AE (Table S3). SAEs were reported in three participants: two (3.4%) in the 50‐mg group (staphylococcal sepsis; Bell's palsy) and one (2.1%) in the 200/50‐mg group (spontaneous abortion). All SAEs were considered unrelated to the study treatment by the investigator. Discontinuation from the study or study drug due to an AE occurred in seven participants (12.1%) in the 50‐mg group (herpes zoster, staphylococcal sepsis, alanine aminotransferase increased, aspartate aminotransferase increased, blood creatine phosphokinase increased, positive interferon gamma release assay, urticaria) and two participants (4.3%) in the 200/50‐mg group (pyrexia and headache in one participant; pregnancy in one participant).

In the 50‐mg group, the 10 most common AEs (occurring in > 5% of participants) were urticaria, pyrexia, upper respiratory tract infection, headache, cough, folliculitis, acne, oropharyngeal pain, SARS‐CoV‐2 test positive, and diarrhea (Table S4). In the 200/50‐mg group, the 10 most common AEs were upper respiratory tract infection, folliculitis, nasopharyngitis, headache, urticaria, pyrexia, urinary tract infection, fall, fatigue, and acne. One participant (1.7%) in the 50‐mg group had a serious infection (staphylococcal sepsis). Herpes zoster was reported in three participants (5.2%) in the 50‐mg treatment group and two participants (4.3%) in the 200/50‐mg treatment group. Herpes simplex was reported in two participants (3.4%) in the 50‐mg treatment group and two participants (4.3%) in the 200/50‐mg treatment group.

No AEs of opportunistic infection, major adverse cardiovascular event, venous thromboembolism, nonmelanoma skin cancer, malignancy excluding nonmelanoma skin cancer, or death were reported.

Leukocyte levels < 0.6× the lower limit of normal (LLN) occurred in one participant (2.1%) in the 200/50‐mg group, and levels > 1.5× the upper limit of normal (ULN) occurred in one participant (2.1%) in the 200/50‐mg group (Table S5). Lymphocyte levels < 0.8 × LLN occurred in nine participants (15.5%) and eight participants (17.0%) in the 50‐mg and 200/50‐mg groups, respectively, and levels > 1.2 × ULN occurred in one participant (2.1%) in the 200/50‐mg group. Neutrophil levels < 0.8 × LLN occurred in two participants (3.4%) and five participants (10.6%) in the 50‐mg and 200/50‐mg groups, respectively, and levels > 1.2 × ULN occurred in two participants (3.4%) and five participants (10.6%) in the 50‐mg and 200/50‐mg groups, respectively. There were no clinically meaningful changes in hemoglobin or platelet parameters in either group. Treatment with ritlecitinib was associated with small decreases in lymphocyte and neutrophil levels in the 50‐mg group and in leukocyte, lymphocyte, and neutrophil levels in the 200/50‐mg group from baseline to week 4 that did not progress over time and remained stable through Month 24. Platelet levels did not decrease in either group. Median changes in hematology parameter levels from baseline in Asian patients were consistent with those in the overall population [19]. Figure S1 shows median changes in lymphocyte levels over time in Asian participants.

Alanine aminotransferase levels > 3× ULN occurred in five participants (8.8%) and one participant (2.1%) in the 50‐mg and 200/50‐mg groups, respectively (Table S6). Two participants (3.4%) had aspartate aminotransferase levels > 3 × ULN, and two participants (3.4%) had bilirubin levels > 1.5 × ULN in the 50‐mg group. There were no clinically meaningful effects of ritlecitinib on aspartate aminotransferase, bilirubin, or cholesterol. Creatine kinase levels > 2 × ULN occurred in 10 participants (17.2%) and seven participants (14.9%) in the 50‐mg and 200/50‐mg groups, respectively; no cases of rhabdomyolysis were reported, and no clinically meaningful change in creatinine levels occurred. Low‐density lipoprotein cholesterol levels > 1.2 × ULN occurred in one participant (2.1%) in the 200/50‐mg group. Triglyceride levels > 1.3 × ULN occurred in two (3.4%) participants in the 50‐mg group. There were no clinically meaningful changes in high‐density lipoprotein cholesterol in either group.

## 4 Discussion

These studies reported the interim efficacy and safety results of treatment with ritlecitinib 50 mg (with or without a 200‐mg loading dose) in participants aged ≥ 12 years with AA in the Asian subpopulation through Month 24. The findings demonstrate the sustained efficacy of up to 2 years of treatment with ritlecitinib in Asian participants. Efficacy results in the Asian subpopulation were consistent with the overall efficacy results from ALLEGRO‐2b/3 [3] and ALLEGRO‐LT up to Month 24 [22]. After 2 years' treatment with ritlecitinib, the majority of Asian participants who achieved a response at Month 12 sustained this response through Month 24. At Month 24, the effects of the loading dose were minimal, with the 200/50‐mg group and the 50‐mg group (who did not receive a loading dose) showing similar levels of sustained efficacy. Together, these results support the long‐term use of ritlecitinib in Asian individuals with AA.

AA is a disease that confers a large psychosocial burden and impact on quality of life [23, 24, 25, 26, 27], with individuals reporting impacts on relationships and work [28, 29, 30]. Additionally, eyebrow and eyelash loss can have a negative impact on an individual's quality of life and can be considered more impactful than scalp hair loss by some [31, 32]. In the current study, eyebrow and eyelash regrowth was reported through up to Month 24, and the proportion of participants with EBA and ELA response among those with abnormal EBA and ELA scores at baseline remained stable through Month 24 in both treatment groups. Additionally, PGI‐C scores of “moderately” or “greatly” improved from baseline were achieved by more than half of participants in each treatment group by Month 12, indicating a correlation between clinical efficacy and participant perceptions of improvement.

Compared to other JAK inhibitors, the mechanism of action of ritlecitinib (selective dual inhibition of JAK3 and TEC family kinases) may have an impact on the safety profile of treatment [20]. In the current study, ritlecitinib was generally well tolerated over 24 months and not associated with any major hematological abnormalities. In the current study, 5.2% and 4.3% of participants in the 50‐mg and 200/50‐mg treatment groups reported herpes zoster infections, compared with 4.2% and 2.6% of participants in the 50‐mg and 200/50‐mg treatment groups in the overall population [19]. Overall, the safety profile in the Asian subpopulation was generally consistent with the overall long‐term safety data for ritlecitinib [20], suggesting that there are no unique risks to Asian participants.

This analysis had some limitations. The number of participants in the Asian subpopulation was small, which limits the generalizability of the results. This is especially the case for safety events that occurred at a lower frequency, such as major adverse cardiovascular events, malignancies, and serious infections. An integrated safety analysis conducted in the overall population, which provides a more robust assessment of the long‐term risk of less‐common events, showed low rates for these safety events and concluded that ritlecitinib had an acceptable safety profile for long‐term use [20]. Additionally, the study only included participants with ≥ 50% hair loss at baseline and those with an episode duration of < 10 years. In general, long‐term open‐label studies, such as ALLEGRO‐LT, may be biased toward those participants who remain in the study, who tolerate the drug, and who show a treatment response.

In conclusion, ritlecitinib showed sustained response in clinician‐assessed scalp hair regrowth, eyebrow and eyelash regrowth, and the participant‐reported outcome, PGI‐C, in Asian participants aged ≥ 12 years with AA. The safety profile in Asian participants is generally consistent with the overall population long‐term safety data. These data support a favorable benefit–risk profile and the long‐term use of ritlecitinib in Asian individuals aged ≥ 12 years with severe AA.

## Funding

This study was sponsored by Pfizer Inc.

## Ethics Statement

The protocol was reviewed and approved by the institutional review boards or ethics committees of the participating institutions. The study was conducted in accordance with the International Ethical Guidelines for Biomedical Research Involving Human Subjects (Council for International Organizations of Medical Sciences 2002), ICH Guideline for Good Clinical Practice, and Declaration of Helsinki. Written informed consent was obtained from each participant or participant's parent or legal representative.

## Conflicts of Interest

R.U. is a clinical trial investigator for Pfizer. M.M. has served as a principal investigator for Eli Lilly Co., Maruho Co., Pfizer Inc., and Sanofi Co and on an advisory board for Kaken Co. K.H. has received lecture fees from Eli Lilly Co. and Bristol Myers Squibb Co.; advisory fees from Eli Lilly Co., Pfizer Inc., and Maruho Co.; and grants/research funds from Shiseido Co., Maruho Co., and Pfizer Inc. At the time of the study, X.L. was an employee of Pfizer R&D China. X.L. is currently an employee of Pfizer Norway and holds stock or stock options in Pfizer. V.B. and R.W. are employees of and hold stock or stock options in Pfizer Inc. M.S.Y. is an employee of Pfizer Japan Inc. and holds stock or stock options in Pfizer Inc. At the time of the study, T.H.'s affiliation was Pfizer Japan Inc. T.H.'s current affiliation is Sun Pharma. N.S. and K.F. are employees of Pfizer R&D GK and hold stock or stock options in Pfizer Inc. Y.S. is a former employee of Pfizer Inc. At the time of the study, Y.S.'s affiliation was Pfizer China PBG. Y.S. does not have a current affiliation. No other conflicts of interest related to this manuscript are declared. X.Z., W.W., W‐.H.C., and O.K. declare no conflicts of interest.

## Supporting information


**Table S1:** Participant disposition in the Asian subpopulation.
**Table S2:** SALT scores at baseline and final visit among participants who completed ALLEGRO 2b‐3 but did not roll over to the ALLEGRO‐LT extension study.
**Table S3:** Summary of treatment‐emergent adverse events.
**Table S4:** Incidence of adverse events that occurred in ≥ 5% of participants in either group and were part of the top 10 most frequent in at least one treatment group.
**Table S5:** Laboratory test abnormalities in leukocyte, neutrophil, lymphocyte, platelet counts, and hemoglobin.
**Table S6:** Laboratory abnormalities for liver tests (ALT, AST, bilirubin, ALP), creatine kinase, and lipids.
**Figure S1:** Lymphocyte median change from baseline in Asian participants.

## Data Availability

Upon request, and subject to review, Pfizer will provide the data that support the findings of this study. Subject to certain criteria, conditions, and exceptions, Pfizer may also provide access to the related individual deidentified participant data. See https://www.pfizer.com/science/clinical‐trials/trial‐data‐and‐results for more information.
