# Supplementary material for: Efficacy and Safety of Ritlecitinib in the Asian Subpopulation of the ALLEGRO‐2b/3 and ALLEGRO‐LT Clinical Studies for Alopecia Areata
Source: J Dermatol. 2026 Feb 6;53(4):578–86. doi: 10.1111/1346-8138.70154 (PMC13075526; doi:10.1111/1346-8138.70154)
Supplement: Supplementary file 1 — Table S1: Participant disposition in the Asian subpopulation. Table S2: SALT scores at baseline and final visit among participants who completed ALLEGRO 2b‐3 but did not roll over to the ALLEGRO‐LT extension study. Table S3: Summary of treatment‐emergent adverse events. Table S4: Incidence of adverse events that occurred in ≥ 5% of participants in either group and were part of the top 10 most frequent in at least one treatment group. Table S5: Laboratory test abnormalities in leukocyte, neutrophil, lymphocyte, platelet counts, and hemoglobin. Table S6: Laboratory abnormalities for liver tests (ALT, AST, bilirubin, ALP), creatine kinase, and lipids. Figure S1: Lymphocyte median change from baseline in Asian participants. [file JDE-53-578-s001.docx]

**Efficacy and safety of ritlecitinib in the Asian subpopulation of the ALLEGRO-2b/3 and ALLEGRO-LT clinical studies for alopecia areata**

**Authors:** Rie Ueki^1^, Masato Mizuashi^2^, Kazutoshi Harada^3^, Xingqi Zhang^4^, Wenyu Wu^5^, Wen-Hung Chung^6^, Ohsang Kwon^7^, Xin Luo^8^, Victoria Basey^9^, Robert Wolk^10^, Nanzhi Shi^11^, Kayo Fujita^11^, Yimeng Shen^12^, Tomohiro Hirose^13^

**Affiliations:** ^1^Juntendo University, Tokyo, Japan; ^2^Tohoku University Graduate School of Medicine, Sendai, Japan; ^3^Department of Dermatology, Tokyo Medical University, Tokyo, Japan; ^4^The First Affiliated Hospital, Sun Yat-sen University, Guangzhou, China; ^5^Huashan Hospital Affiliated to Fudan University, Shanghai, China; ^6^Department of Dermatology, Chang Gung Memorial Hospital, Taipei, Taiwan; ^7^Department of Dermatology, Seoul National University College of Medicine, Seoul, South Korea; ^8^Pfizer R&D China, China; ^9^Pfizer Inc, UK; ^10^Pfizer Inc, Groton, CT, USA; ^11^Pfizer R&D Japan G.K., Japan; ^12^Pfizer China PBG, Beijing, China; ^13^ Pfizer Japan Inc, Japan

**Corresponding author:**

Victoria Basey

Pfizer Inc, UK

Email: victoria.basey@pfizer.com

# APPENDIX

**Table S1.** Participant disposition in the Asian subpopulation

| **Disposition, n (%)** | **Ritlecitinib**  **50 mg**  **(n=58)** | **Ritlecitinib**  **200/50 mg**  **(n=47)** |
| --- | --- | --- |
| Ongoing at the data cutoff date^†^ | 37 (63.8) | 34 (72.3) |
| Completed ALLEGRO 2b-3 study but did not roll over to extension study | 7 (12.1) | 5 (10.6) |
| Discontinued | 14 (24.1) | 8 (17.0) |
| Adverse event | 7 (12.1) | 1 (2.1) |
| Lack of efficacy | 1 (1.7) | 1 (2.1) |
| Pregnancy | 0 | 1 (2.1) |
| Withdrawal by participant | 3 (5.2) | 3 (6.4) |
| No longer met eligibility criteria^‡^ | 1 (1.7) | 2 (4.3) |
| Other | 2 (3.4) | 0 |

^†^December 9, 2022.
^‡^The 1 participant in the 50-mg group and 2 participants in the 200/50-mg group no longer met the continuation criteria for adolescents. Continuation criteria for adolescents (age 12-17 years) in ALLEGRO-LT required ≥50% improvement in Severity of Alopecia Tool (SALT) score by Month 3 for rollover participants from ALLEGRO-2b/3 and a SALT score of ≤20 by Month 6 in ALLEGRO-LT. If participants discontinued due to not meeting the continuation criteria, the discontinuation reason was categorized as “no longer meets eligibility criteria.”

**Table S2.** SALT scores at baseline and final visit among participants who completed ALLEGRO 2b-3 but did not roll over to the ALLEGRO-LT extension study

| **Group** | **Patient ID** | **Baseline SALT score** | **Last SALT score in the last visit of the ALLEGRO 2b/3 study** | **Last SALT ≤20 response status in the last visit of the ALLEGRO 2b/3 study** |
| --- | --- | --- | --- | --- |
| ritlecitinib 200/50 mg | 1 | 73.9 | 0.0 | Responder |
|  | 2 | 58.0 | 3.0 | Responder |
|  | 3 | 63.3 | 13.1 | Responder |
|  | 4 | 99.0 | 87.1 | Non- Responder |
|  | 5 | 100.0 | 91.2 | Non- Responder |
| ritlecitinib 50 mg | 1 | 83.6 | 0.0 | Responder |
|  | 2 | 83.3 | 0.0 | Responder |
|  | 3 | 56.2 | 0.0 | Responder |
|  | 4 | 100.0 | 18.1 | Responder |
|  | 5 | 77.7 | 44.0 | Non- Responder |
|  | 6 | 100.0 | 70.9 | Non- Responder |
|  | 7 | 100.0 | 100.0 | Non- Responder |

SALT, Severity of Alopecia Tool.

“Responder” was defined as patient achieving SALT score of ≤20.

**Table S3.** Summary of treatment-emergent adverse events

| **n (%)** | **Ritlecitinib**  **50 mg**  **(n=58)** | **Ritlecitinib**  **200/50 mg**  **(n=47)** |
| --- | --- | --- |
| Participants with adverse events | 57 (98.3) | 44 (93.6) |
| Participants with serious adverse events | 2 (3.4) | 1 (2.1) |
| Participants discontinued from study or from study drug due to adverse events | 7 (12.1) | 2 (4.3) |

Safety data are presented through to the time of data cutoff, December 9, 2022.

**Table S4.** Incidence of adverse events that occurred in ≥5% of participants in either group and were part of the top 10 most frequent in at least one treatment group

| **n (%)** | **Ritlecitinib**  **50 mg**  **(n=58)** | **Ritlecitinib**  **200/50 mg**  **(n=47)** |
| --- | --- | --- |
| Urticaria | 11 (19.0) | 7 (14.9) |
| Pyrexia | 11 (19.0) | 5 (10.6) |
| Upper respiratory tract infection | 10 (17.2) | 13 (27.7) |
| Headache | 10 (17.2) | 8 (17.0) |
| Cough | 10 (17.2) | 1 (2.1) |
| Folliculitis | 8 (13.8) | 13 (27.7) |
| Acne | 8 (13.8) | 2 (4.3) |
| Oropharyngeal pain | 8 (13.8) | 1 (2.1) |
| SARS-CoV-2 test positive | 8 (13.8) | 1 (2.1) |
| Diarrhea | 7 (12.1) | 1 (2.1) |
| Nasopharyngitis | 6 (10.3) | 9 (19.1) |
| Fatigue | 5 (8.6) | 3 (6.4) |
| Urinary tract infection | 2 (3.4) | 5 (10.6) |
| Fall | 2 (3.4) | 4 (8.5) |

Safety data are presented through to the time of data cutoff, December 9, 2022.

**Table S5**. Laboratory test abnormalities in leukocyte, neutrophil, lymphocyte, platelet counts, and hemoglobin

| **n (%)** | **Ritlecitinib**  **50 mg**  **(n=58)** | **Ritlecitinib**  **200/50 mg**  **(n=47)** |
| --- | --- | --- |
| Leukocytes (×10^3^/μL)  <0.6 × LLN  >1.5 × ULN | 0  0 | 1 (2.1)  1 (2.1) |
| Lymphocytes (×10^3^/μL)  <0.8 × LLN  >1.2 × ULN | 9 (15.5)  0 | 8 (17.0)  1 (2.1) |
| Neutrophils (×10^3^/μL)  <0.8 × LLN  >1.2 × ULN | 2 (3.4)  2 (3.4) | 5 (10.6)  5 (10.6) |
| Platelets (×10^3^/μL)  <0.5 × LLN  >1.75 × ULN | 0  0 | 0  0 |
| Hemoglobin (g/dL) <0.8 × LLN | 0 | 0 |

LLN, lower limit of normal; ULN, upper limit of normal.
Safety data are presented through to the time of data cutoff, December 9, 2022.

**Table S6.** Laboratory abnormalities for liver tests (ALT, AST, bilirubin, ALP), creatine kinase, and lipids

| **n (%)** | **Ritlecitinib**  **50 mg**  **(n=58)** | **Ritlecitinib**  **200/50 mg**  **(n=47)** |
| --- | --- | --- |
| ALT (U/L) >3.0 × ULN | 5 (8.6) | 1 (2.1) |
| AST (U/L) >3.0 × ULN | 2 (3.4) | 0 |
| Bilirubin (mg/dL) >1.5× ULN | 2 (3.4) | 0 |
| ALP (U/L) >3.0 × ULN | 0 | 0 |
| Creatine kinase (U/L) >2.0 × ULN | 10 (17.2) | 7 (14.9) |
| HDL cholesterol (mg/dL) <0.8 × LLN | 0 | 0 |
| LDL cholesterol (mg/dL) >1.2 × ULN | 0 | 1 (2.1) |
| Triglycerides (mg/dL) >1.3 × ULN | 2 (3.4) | 2 (4.3) |

ALT, alanine aminotransferase; ALP, alkaline phosphatase; AST, aspartate aminotransferase; HDL, high-density lipoprotein; LDL, low-density lipoprotein; LLN, lower limit of normal.

Safety data are presented to data cutoff, December 9, 2022.

**Figure S1.** Lymphocyte median change from baseline in Asian participants


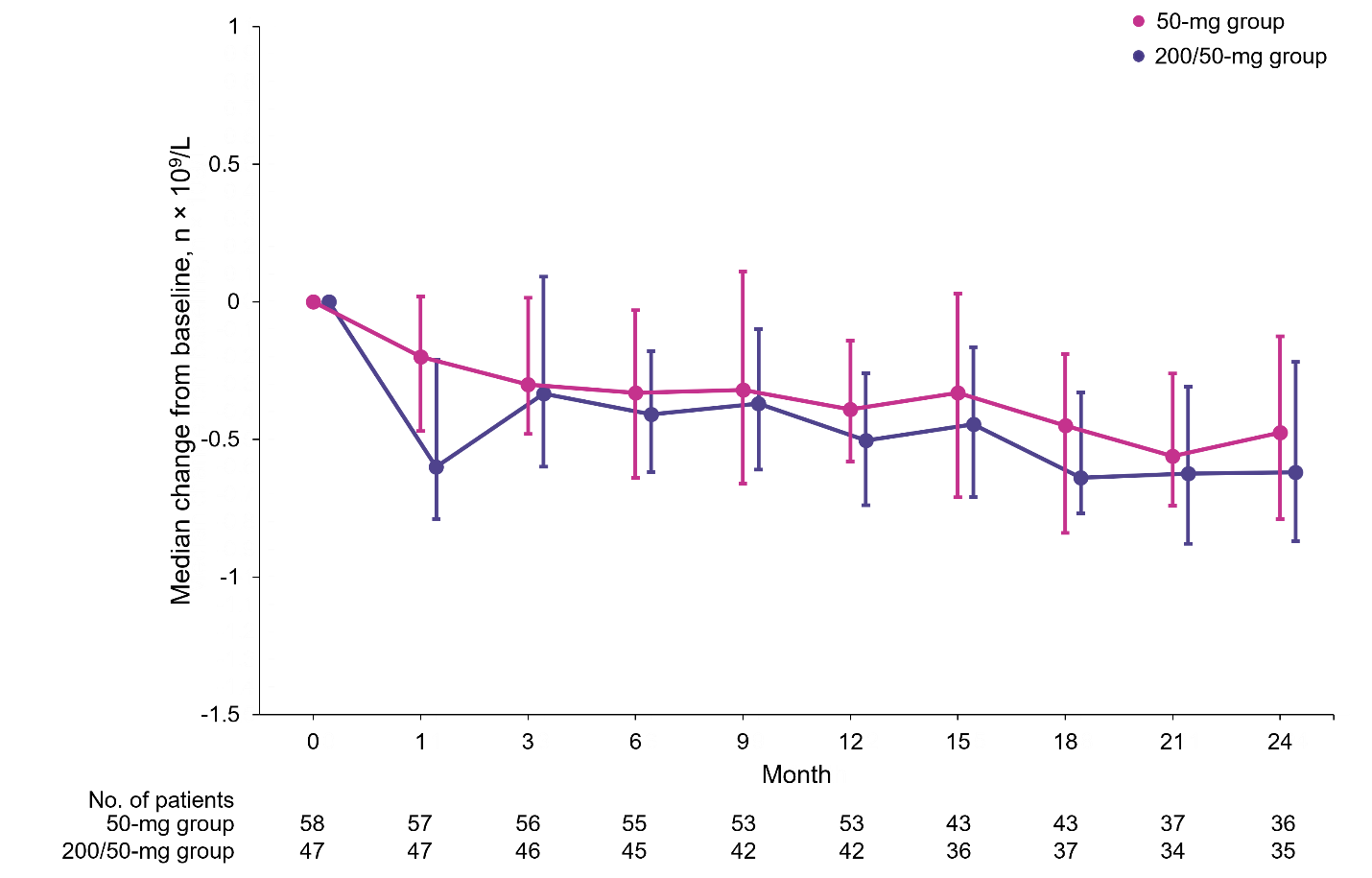


Week 0 corresponds to baseline. Error bars represent Q1-Q3.
